# Identification and validation of a risk assessment scoring tool for extended-spectrum beta-lactamase-producing Enterobacterales bacteremia at a tertiary teaching hospital

**DOI:** 10.1017/ash.2025.70

**Published:** 2025-04-24

**Authors:** Victoria Gavaghan, Jessica L. Miller, Maureen Shields, Jennifer Dela-Pena

**Affiliations:** 1 Department of Pharmacy, Advocate Lutheran General Hospital, 1775 Dempster Street, Park Ridge, IL 60068, USA; 2 Advocate Aurora Research Institute, 945 N 12^th^ Street, Milwaukee, WI 53233, USA; 3 Department of Pharmacy, Cleveland Clinic, 9500 Euclid Avenue, Cleveland, OH 44195, USA

## Abstract

**Objective:**

To identify institution-specific risk factors for extended-spectrum beta-lactamase (ESBL) bloodstream infections (BSI) to develop and validate a risk assessment scoring tool that can be utilized for hospitalized patients.

**Design:**

Single-center, retrospective, case-control study.

**Setting:**

Tertiary teaching hospital.

**Patients:**

Hospitalized adult and pediatric patients with *E. coli* or *Klebsiella* spp. BSI were stratified based on ESBL production between August 2019 to July 2021. Exclusion criteria included patients < 28 days old, a positive blood culture resulting prior to admission/after discharge or a polymicrobial and/or carbapenem-resistant BSI.

**Methods:**

Multivariable logistic regression assessed predictors of ESBL in a derivation cohort. Predictors were applied to a novel validation BSI cohort using area under the receiver-operator characteristics curve (ROC AUC) to assess the reliability of identifying patients likely to harbor ESBL at the time of organism identification.

**Results:**

A total of 238 patients in the derivation cohort met inclusion criteria stratified as ESBL (n = 68) or non-ESBL (n = 170). Multivariable logistic regression demonstrated diabetes, 30-day history of invasive procedure or antibiotic use, and/or history of ESBL as independent predictors of ESBL. After creation of an ESBL risk assessment tool, the results were applied to a validation cohort of 170 patients. This model displayed good calibration and discrimination with a strong predictive power (Hosmer-Lemeshow χ^2^= 4.66, p = 0.19; ROC AUC = 0.88, 95% CI = 0.7909 – 0.974).

**Conclusions:**

A validated ESBL risk assessment tool reliably identified hospitalized patients likely to harbor ESBL *E. coli* or *Klebsiella* spp. BSI upon organism identification.

## Introduction

Extended-spectrum beta-lactamase (ESBL) bloodstream infections (BSI) contribute to increased morbidity, mortality, and healthcare costs.^1–4^ In 2017, an estimated 197,400 cases of ESBL-producing Enterobacterales infections among hospitalized patients were reported, resulting in 9,100 deaths in the United States with rates steadily increasing since 2012.^2^ Carbapenems remain the treatment of choice for ESBL BSI. However, increased empiric carbapenem use has spurred the development of carbapenem resistance, prompting the need for ESBL risk factor assessment tools.^2,5–10^ Early recognition of patients at risk for ESBL BSI can help optimize empiric antimicrobial therapy while avoiding unnecessary use of carbapenems for low-risk patients.

Risk factors for ESBL include prolonged stay in hospital wards, nursing homes, or intensive care units, prior ß-lactam or fluoroquinolone use, and invasive procedures such as placement of indwelling catheters or mechanical ventilation.^6,8,10–13^ However, risk factors may be institution-specific based on the local patient population.

Devising and implementing a validated, predictive risk score model for ESBL BSI may help with clinical decision-making. Previous studies assessing the use of an ESBL clinical prediction tool have shown reliability in stratifying low and high-risk patients and may prove useful in tailoring empiric therapy.^14–21^ The aim of our study was to identify institution-specific risk factors for ESBL BSI in order to develop a validated scoring tool that predicts the clinical risk of ESBL for hospitalized patients presenting with either *E. coli* or *Klebsiella* spp. BSI.

## Methods

### Study design and population

This was a single-center, retrospective, case-control study conducted at Advocate Lutheran General Hospital, a 670-bed tertiary teaching hospital located in Park Ridge, Illinois, USA. Adult and pediatric patients were enrolled in a derivation cohort if they were hospitalized and received antimicrobial therapy for a documented *E. coli* or *Klebsiella* spp. BSI between August 1, 2019 and June 30, 2020. Patients meeting inclusion criteria were further stratified based on ESBL production. Patients were excluded if they were < 28 days old, their positive blood culture resulted prior to admission or after discharge or had polymicrobial and/or carbapenem-resistant BSI. Patients with documented *E. coli* and *Klebsiella spp.* BSI were enrolled in a validation cohort between July 1, 2020 and August 30, 2021. Manual chart review was performed to collect demographic information, comorbidities, microbiologic and antimicrobial history, hospitalization information, and clinical outcomes. Study data were collected and managed using REDCap, a password-protected electronic data capture tool hosted by Advocate Health.^22,23^ This study was exempt from institutional review board oversight.

### Outcomes

The primary outcome was determination of institution-specific risk factors associated with ESBL BSI to develop and validate an ESBL BSI scoring tool.

### Definitions

Community-onset BSI was defined as positive blood culture result occurring <48 hours after hospital admission. Utilization of immunosuppressive agents was defined as receipt of calcineurin inhibitors, antimetabolites, or chemotherapy within the past 90 days, and/or corticosteroid usage at doses >20 mg of prednisone or equivalent for ≥2 weeks.^24^ All available laboratory parameters were assessed for the largest derangement from normal limits within 24 hours of positive blood culture result. Surgery or invasive procedures included any of the following: any type of surgery, insertion of central venous catheters, nasogastric/gastrostomy-jejunostomy tubes, foley catheters, drainage tubes, urinary stent, or tracheostomy/mechanical ventilation. When assessing history of ESBL, no differentiation was made between infection versus colonization.

### Microbiology methods

Identification of organisms and susceptibility testing were performed at the institutional microbiology lab (ACL Laboratories). Organism identification was performed by MALDI-TOF mass spectrometry (Vitek MS, bioMérieux, https://www.biomerieux.com). Susceptibilities were interpreted using the Clinical and Laboratory Standards Institute breakpoints.^25^ Phenotypic testing for ESBL was performed on *E. coli*, *K. pneumoniae,* and *K. oxytoca* using the AST-GN69 VITEK 2 Gram Negative Susceptibility Card (bioMérieux, Durham, NC) on a VITEK 2 automated bacterial identification and susceptibility testing instrument (bioMérieux). By means of double-disk diffusion, the AST-GN69 test card and the VITEK 2 instrument simultaneously assess the inhibitory effects of cefepime, cefotaxime, and ceftazidime alone and in combination with clavulanic acid on the index isolate to determine if ESBL was likely to be present. Confirmatory testing using alternative methods for ESBL detection, such as combination disc methods or E-test ESBL strips, was not performed.

### Statistical methods

Univariate and bivariate analyses were performed to evaluate the distribution of the derivation cohort and to compare ESBL and non-ESBL groups. Continuous variables were compared using the Student’s t-test for normally distributed variables and Mann-Whitney U test for non-normally distributed variables. Categorical variables were compared using the Chi-Square Test or Fisher’s Exact Test for cell counts <5. Measures of association include corresponding odds ratios and 95% confidence intervals. A multivariable logistic regression was conducted to identify risk factors associated with ESBL production while controlling for confounders and adjusting for outliers. Variables were included in the model based on a combination of statistical significance (*P* < 0.05) in the bivariate analysis and clinical importance. A manual model-building process was used to evaluate estimates and verify the importance of each predictor to include in the final model. A Firth regression was run on the final model to address a wide confidence interval due to the likely presence of separation. Statistical significance was established by a two-tailed alpha of 0.05.

Upon identification of risk factors associated with ESBL production, a scored assessment tool was developed to predict risk of ESBL BSI using the beta/integer scheme.^26^ The scored tool was applied to both a derivation and validation cohort. To validate the prediction score, the area under the receiver-operator characteristics curve (ROC AUC) was assessed for both cohorts. Sensitivity, specificity, positive predictive value (PPV), and negative predictive value (NPV), along with their 95% confidence intervals, were calculated for each cutoff value. The Hosmer-Lemeshow test for goodness-of-fit was used to assess calibration among both cohorts.^27^ All analyses were performed using SAS statistical software (Version 9.4; SAS Institute, Cary, NC).

## Results

### Baseline characteristics

*(i) Derivation cohort*: A total of 238 patients met inclusion criteria, 68 with ESBL BSI and 170 with non-ESBL BSI. Most patients in the overall cohort were of white ethnicity (57, 83.8% versus 133, 78.2%), ≥65 years of age (51, 75% versus 118, 69.4%), and immunocompetent (61, 89.7% versus 153, 90%). Patients with ESBL BSI were more likely to present from a skilled nursing facility (21, 30.9% versus 32, 18.8% *P* = 0.04) and have a history of diabetes (36, 52.9% versus 60, 35.3% *P* = 0.01). Otherwise, no significant differences were observed in baseline characteristics (Table 1). Among the total cohort, the most common organisms isolated were *E. coli* (184, 77.3%) and *K. pneumoniae* (44, 18.5%). Urinary tract (169, 71%) and intra-abdominal (54, 22.7%) were the most common infection sources. No differences were observed between groups for organism isolation and infection source. Infections were more likely to be community-onset (198, 83.2%) with index culture draw occurring in the emergency department (51, 75% versus 150, 88.2% *P* = 0.03). Most patients were admitted to a general nursing floor (41, 80.4% versus 116, 77.3%), and patients with ESBL BSI had a significantly longer hospital length of stay (13 d versus 8.8 d *P* = 0.04). No other differences in laboratory parameters at time of blood culture draw or patient disposition were observed (Table 2).

**Table 1.** Baseline characteristics and laboratory parameters – derivation cohort

Parameter	ESBL; N = 68	Non-ESBL; N = 170	P-value
Age (years), mean (±SD)	73.0 (±13.2)	72.9 (±15.9)	0.71
<18 yr	0 (0)	2 (1.2)	
18–64 yr	17 (25)	50 (29.4)	
≥65 yr	51 (75)	118 (69.4)	
Male, n (%)	30 (44.1)	86 (50.6)	0.37
Ethnicity, n (%)			0.59
African American	1 (1.5)	2 (1.2)	
Asian	4 (5.9)	19 (11.2)	
Hispanic/Latino	5 (7.4)	15 (8.8)	
White	57 (83.8)	133 (78.2)	
Other	1 (1.5)	1 (0.6)	
Smoking History, n (%)	23 (33.8)	38 (22.4)	0.07
ETOH History, n (%)	4 (5.9)	10 (5.9)	>0.99
Trauma History, n (%)	43 (63.2)	90 (52.9)	0.15
Skilled Nursing Facility, n (%)	21 (30.9)	32 (18.8)	0.04
Diet, n (%)			0.65
NPO	5 (7.4)	9 (5.3)	
TPN	2 (2.9)	2 (1.2)	
Tube feeds	4 (5.9)	12 (7.1)	
General Diet	57 (83.8)	147 (86.5)	
CCI score, mean (±SD)	5.3 (±2.2)	4.8 (±2.4)	0.12
APACHE II score, mean (±SD)	16 (±5.4)	14.9 (±8.4)	0.24
PITT Bacteremia score, mean (±SD)	1.5 (±1.4)	1.6 (±2.0)	0.91
Immunocompromised, n (%)	7 (10.3)	17 (10)	0.95
Type, n (%)			
Transplant Medication	2 (2.9)	4 (2.4)	
Chemotherapy	6 (8.8)	14 (8.2)	0.88
Steroid	0 (0)	4 (2.4)	0.58
Transplant Medication, n (%)			
CNI	2 (2.9)	2 (1.2)	0.32
Antimetabolite	1 (1.5)	3 (1.8)	1
mTOR inhibitor	1 (1.5)	1 (0.6)	0.49
Comorbidities, n (%)			
Diabetes	36 (52.9)	60 (35.3)	0.01
Hypertension	48 (70.6)	113 (66.5)	0.54
Benign prostatic hyperplasia	7 (10.3)	28 (16.5)	0.22
Structural biliary/urinary	6 (8.8)	5 (2.9)	0.08
Chronic heart disease	16 (23.5)	31 (18.2)	0.35
Liver disease, no cirrhosis	0 (0)	1 (0.6)	1
Liver disease, cirrhosis	4 (5.9)	5 (2.9)	0.28
COPD	13 (19.1)	19 (11.2)	0.1
CKD, no dialysis	10 (14.7)	24 (14.1)	0.91
CKD, dialysis	3 (4.4)	6 (3.5)	0.72
Cancer, metastatic	3 (4.4)	11 (6.5)	0.76
Cancer, non-metastatic	12 (17.7)	25 (14.7)	0.57
Hematologic malignancy	6 (8.8)	11 (6.5)	0.52
Solid tumor	13 (19.1)	36 (21.2)	0.72
HIV/AIDS	1 (1.5)	0 (0)	0.29
SARS-CoV-2 positive	3 (4.4)	2 (1.2)	0.14

*AIDS: acquired immunodeficiency syndrome; CCI: Charlson comorbidity index; COPD: chronic obstructive pulmonary disease; CKD: chronic kidney disease; ETOH: alcohol abuse; HIV: human immunodeficiency virus; NPO: nothing by mouth; TPN: total parenteral nutrition

^¥^For patients who qualified for multiple factors, patients were counted for each category they met.

**Table 2.** Hospital admission data – derivation cohort

Parameter	ESBL; N = 68	Non-ESBL; N = 170	P-value
Hospital location index culture, n (%)			0.03
ED	51 (75)	150 (88.2)	
Floor	13 (19.1)	16 (9.4)	
ICU	4 (5.9)	4 (2.4)	
Admission Disposition, n (%)			0.65
Floor	41 (80.4)	116 (77.3)	
ICU	10 (19.6)	34 (22.7)	
ICU at the time of index culture, n (%)	7 (12.9)	13 (9.9)	0.53
ICU days prior to culture, mean (±SD)	2.2 (±3.2)	12.4 (±18.5)	0.35
ICU LOS^⁑^, mean (±SD)	4.5 (±5.9)	4.8 (±8.2)	0.89
Hospital LOS, mean (±SD)	13.0 (±8.9)	8.8 (±9.4)	0.04
CRP (mg/L), mean (±SD)	18.9 (± 8.4)	12.9 (±9.6)	0.07
WBC (cells/L), mean (±SD)	15.7 (±7.7)	14.8 (±8.2)	0.42
Temperature (^o^C), mean (±SD)	38.4 (±1.2)	38.0 (±2.9)	0.21
Procalcitonin (ng/mL), mean (±SD)	32.0 (±55.0)	23.7(±63.4)	0.54
Organism, n (%)			
*E. coli*	61 (89.7)	123 (72.3)	0.001
*K. pneumoniae*	7 (10.3)	37 (21.8)	0.04
*K. oxytoca*	0 (0)	10 (5.9)	0.07
Onset of infection, n (%)			0.001
Community	49 (72.1)	149 (87.7)	
Nosocomial	19 (27.9)	21 (12.4)	
Type of infection, n (%)			
Bone and Joint SSTI	1 (1.5) 0 (0)	0 (0) 1 (0.6)	0.29 1
Respiratory	1 (1.5)	6 (3.5)	0.68
Urinary	51 (75)	118 (69.4)	0.39
Intraabdominal	13 (19.1)	41 (24.1)	0.41
Catheter/Line	2 (2.9)	1 (0.6)	0.19
Unknown	2 (2.9)	3 (1.8)	0.63
Other	1 (1.5)	3 (1.8)	1

*CRP: c-reactive protein; ED: emergency department; ICU: intensive care unit; LOS: length of stay; SSTI: Skin and soft tissue infection; WBC: white blood cell count

^⁑^Donated only for patients admitted into the intensive care unit.

^¥^For patients who qualified for multiple factors, patients were counted for each category they met.

Patients presenting with ESBL BSI were more likely to have a prior hospitalization in the previous 90 days (34, 50% versus 54, 31.8% *P* = 0.01), prior procedure in the past 30 days (32, 47.1% versus 31, 18.2% *P* < 0.0001) and 90 days (37, 54.4% versus 48, 28.4% *P* < 0.0002), and history of antibiotic use in the past 30 (41, 60.3% versus 43, 25.3% *P* < 0.0001) and 90 days (47, 69.1% versus 60, 35.3% *P* < 0.0001). A detailed breakdown of previous antibiotic exposure is in Table 3. Among patients with a history of prior procedure, those presenting with ESBL BSI were more likely to have a history of urinary catheter insertion (19, 27.9% versus 16, 9.4% *P* = 0.0003) or surgical procedure (16, 23.5% versus 18, 10.6% *P* = 0.01) with no differences observed for other invasive procedures. There was a statistically significant difference between groups for all-time history of ESBL-producing organism (23, 33.8% versus 1, 0.6% *P* < 0.0001). A multivariate analysis demonstrated that diabetes (OR 2.19; 95% CI 1.09–4.39), prior procedure in the previous 30 days (OR 2.61; 95% CI 1.22–5.59), history of antibiotic usage in the previous 30 days (OR 2.84; 95% CI 1.39–5.79), and history of ESBL infection regardless of timeframe (OR 38.19; 95% CI 6.82–213.78) were independent risk factors for the emergence of ESBL BSI (Table 4).

**Table 3.** Univariate analysis of clinical characteristics – derivation cohort

Parameter	ESBL; N = 68	Non-ESBL; N = 170	P-value
Prior hospitalization, 30 d, n (%)	18 (26.5)	33 (19.4)	0.23
Prior hospitalization, 90 d, n (%)	34 (50)	54 (31.8)	0.01
Prior ICU, 30 d, n (%)	6 (8.8)	7 (4.1)	0.15
Prior ICU, 90 d, n (%)	9 (13.2)	12 (7.1)	0.14
Prior procedure, 30 d, n (%)	32 (47.1)	31 (18.2)	<0.0001
Prior procedure, 90 d, n (%)	37 (54.4)	48 (28.4)	<0.0002
Procedure, n (%)			
Mechanical Ventilation	2 (2.9)	5 (2.9)	1
Tracheostomy	2 (2.9)	2 (1.2)	0.33
NG/JG Tube Insertion	6 (8.8)	8 (4.7)	0.23
Urinary Catheter	19 (27.9)	16 (9.4)	0.0003
Central Line	7 (10.3)	10 (5.9)	0.23
Drainage Tube	6 (8.8)	9 (5.3)	0.31
Surgery	16 (23.5)	18 (10.6)	0.01
Stent Insertion	7 (10.3)	8 (4.7)	0.11
History of Antibiotics, 30 d, n (%)	41 (60.3)	43 (25.3)	<0.0001
History of Antibiotics, 90 d, n (%)	47 (69.1)	60 (35.3)	<0.0001
Antibiotic History; Type, n (%)			
Aminopenicillins	1 (1.5)	4 (2.4)	1
Cephalosporins	22 (32.4)	32 (18.8)	0.02
Fluoroquinolones	14 (20.6)	11 (6.5)	0.001
BLBLI	13 (19.1)	15 (8.8)	0.03
Carbapenems	12 (17.7)	6 (3.5)	0.0002
Nitrofurantoin	3 (4.4)	4 (2.4)	0.41
Trimethoprim/Sulfamethoxazole	10 (14.7)	6 (3.5)	0.002
Fosfomycin	1 (1.5)	0 (0)	0.29
Tetracycline	0 (0)	2 (1.2)	1
Aminoglycosides	1 (1.5)	0 (0)	0.29
Cephalosporin generation, n (%)			
1^st^	9 (13.2)	11 (6.5)	0.09
2^nd^	0 (0)	1 (0.6)	1
3^rd^	11 (16.2)	10 (5.9)	0.01
4^th^	7 (10.3)	16 (9.4)	0.84
Route of Antibiotics, n (%)			
Enteral	28 (41.2)	39 (22.9)	0.0047
Intravenous	28 (41.2)	29 (17.1)	<0.0001
History of ESBL, anytime, n (%)	23 (33.8)	1 (0.6)	<0.0001
History of ESBL, 30 d, n (%)	10 (4.2)	1 (0.4)	<0.0001
History of ESBL, 90 d, n (%)	17 (7.1)	1 (0.4)	<0.0001

*BLBLI: beta lactam beta lactamase inhibitor; ESBL: extended-spectrum beta lactamase; ICU: intensive care unit; JG: gastrostomy-jejunostomy; NG: nasogastric

^¥^For patients who qualified for multiple factors, patients were counted for each category they met.

**Table 4.** Multivariate logistic regression analysis assessing predictors of ESBL infection with corresponding point values – derivation cohort

Parameter	β-coefficient	Odds Ratio (95% CI)	P-value	Score
Diabetes	0.78	2.19 (1.09–4.39)	0.03	1
Prior Procedure, 30 d	0.96	2.61 (1.22–5.59)	0.01	1
History of Antibiotics, 30 d	1.04	2.84 (1.39–5.79)	<0.01	1
History of ESBL, anytime	3.64	38.19 (6.82–213.78)	<0.01	4

ESBL, extended-spectrum beta lactamase

*(ii) Validation Cohort:* From July 2020 through August 2021 a total of 170 patients were included in the validation cohort, 29 (17.1%) were ESBL. Complete baseline characteristics were not collected for this cohort. Instead, risk factors from the derivation cohort found to be independent predictors of ESBL were applied. Compared with the derivation cohort, those with ESBL BSI in the validation cohort had a lower incidence of diabetes (10, 34.5%) and prior procedure in the past 30 days (12, 41.4%) but a higher incidence of antibiotic usage in the previous 30 days (21, 72.4%) and all-time history of ESBL infection (19, 65.5%).

### Construction and validation of a predictive scoring tool

**Figure 1.**
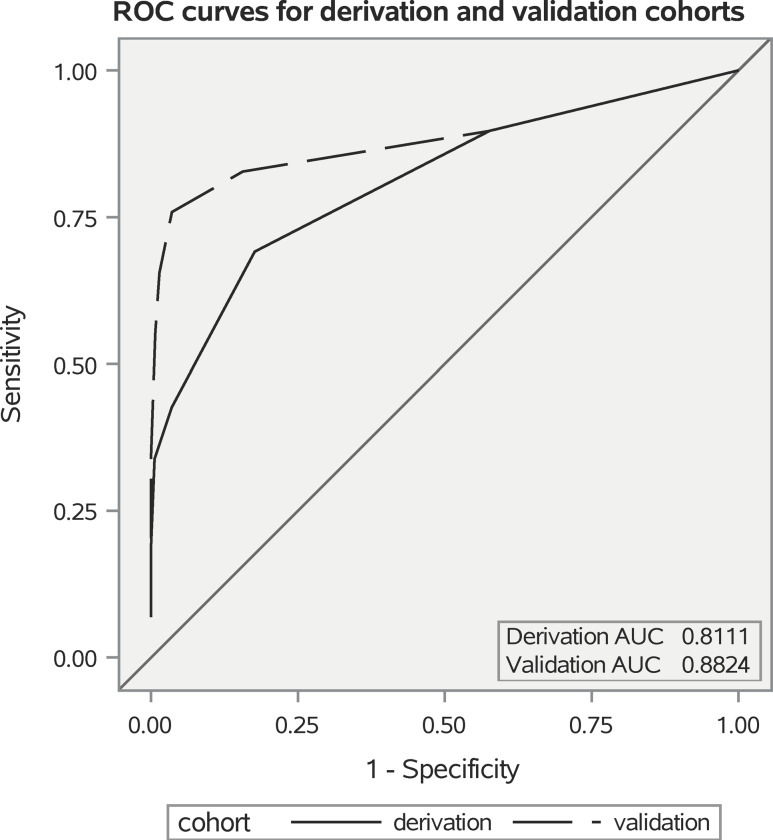
Receiver-operator characteristics curves - derivation and validation cohorts .

*(i) Derivation cohort*: A weighted score was assigned to each risk factor based on results from the multivariate analysis for a maximum score of 7 (Table 4). The distribution of overall scores between ESBL BSI and non-ESBL BSI is summarized in Table 5. Scores of 0 and 1 were found primarily in non-ESBL BSI while scores of 6 and 7 were found exclusively in ESBL BSI. The ROC AUC was 0.81 (95% CI 0.7477–0.8745), which indicates good discrimination performance of the model suggesting that it performs well in discriminating risk at both validations sets (Figure 1). The results of the Hosmer-Lemshow chi-squared testing (χ^2^ = 1.19, *P* = 0.754) suggest good calibration of the proposed scoring tool.

**Table 5.** Distribution of overall scores in the risk assessment tool - derivation and validation cohorts

Score	Derivation Cohort			Validation Cohort		
	ESBL; N (%)	Non-ESBL; N (%)	Total; N	ESBL; N (%)	Non-ESBL; N (%)	Total; N
**0**	7 (8.7)	72 (91.1)	79	3 (4.8)	60 (95.2)	63
**1**	14 (17.1)	68 (82.9)	82	2 (3.3)	59 (96.7)	61
**2**	18 (42.6)	24 (57.1)	42	2 (10.5)	17 (89.5)	19
**3**	6 (54.5)	5 (45.5)	11	3 (50)	3 (50)	6
**4**	0	0	0	3 (75)	1 (25)	4
**5**	10 (90.9)	1 (9.1)	11	6 (85.7)	1 (14.3)	7
**6**	8 (100)	0 (0)	8	8 (100)	0	8
**7**	5 (100)	0 (0)	5	2 (100)	0	2
**Total**	68 (28.6)	170 (71.4)	238	29 (17.1)	141 (82.9)	170

ESBL, extended-spectrum beta lactamase

As demonstrated by the risk score performance (Table 6), a low cutoff displayed high sensitivity but lost specificity whereas a high cutoff significantly increased specificity but inversely impacted sensitivity. Defining high-risk ESBL BSI as a score of ≥3, the sensitivity was low (42.7%), while retaining excellent specificity (96.5%). The positive predictive value (PPV) and negative predictive value (NPV) had an inverse relationship with a decrease in PPV and an increase in NPV with a higher calculated risk score. The cutoff level of ≥3 was associated with a PPV and NPV of 82.9% and 80.8%, respectively.

**Table 6.** Risk score performance – derivation and validation cohorts

Derivation Cohort				
Score	Sensitivity (95% CI)	Specificity (95% CI)	PPV (95% CI)	NPV (95% CI)
≥7	0.07 (0.01–0.14)	1 (1.0–1.0)	1 (1.0–1.0)	0.73 (0.67–0.79)
≥6	0.19 (0.09–0.28)	1 (1.0–1.0)	1 (1.0–1.0)	0.76 (0.69–0.81)
≥5	0.34 (0.23–0.45)	0.99 (0.98–1.0)	0.96 (0.88–1.0)	0.79 (0.74–0.85)
≥4	0.34 (0.23–0.45)	0.99 (0.98–1.0)	0.96 (0.88–1.0)	0.79 (0.74–0.85)
≥3	0.43 (0.31–0.54)	0.96 (0.94–0.99)	0.83 (0.70–0.95)	0.81 (0.75–0.86)
≥2	0.69 (0.58–0.80)	0.82 (0.77–0.88)	0.61(0.31–0.46)	0.87 (0.82–0.92)
≥1	0.89 (0.82–0.97)	0.42 (0.35–0.49)	0.38 (0.31–0.46)	0.91 (0.85–0.97)
**Validation Cohort**				
**Score**	**Sensitivity (95% CI)**	**Specificity (95% CI)**	**PPV (95% CI)**	**NPV (95% CI)**
≥7	0.07 (0–0.16)	1 (1.0-1.0)	1 (1.0-1.0)	0.84 (0.78–0.89)
≥6	0.34 (0.17–0.52)	1 (1.0-1.0)	1 (1.0-1.0)	0.88 (0.83–0.93)
≥5	0.55 (0.37–0.73)	0.99 (0.98–1.0)	0.94 (0.83–1.0)	0.92 (0.87–0.96)
≥4	0.66 (0.48–0.83)	0.99 (0.97–1.0)	0.91 (0.78–1.0)	0.93 (0.89–0.97)
≥3	0.76 (0.60–0.91)	0.96 (0.67–0.96)	0.81 (0.67–0.96)	0.95 (0.92–0.99)
≥2	0.83 (0.69–0.97)	0.84 (0.78–0.90)	0.52 (0.38–0.67)	0.96 (0.93–0.99)
≥1	0.89 (0.79–1.0)	0.43 (0.34–0.51)	0.24 (0.16–0.32)	0.95 (0.89–1.0)

*(ii) Validation cohort*: The distribution of overall scores of ESBL BSI compared to non-ESBL BSI is summarized in Table 5. Similarly, as in the derivation cohort, scores of 0, 1, and 2 were primarily associated with non-ESBL BSI while scores of 6 and 7 were found exclusively in ESBL BSI. As demonstrated in Figure 1, when the ESBL prediction tool was applied, it exhibited a strong predictive power (ROC AUC = 0.88, 95% CI = 0.7909 – 0.9741) and a good calibration (Hosmer-Lemeshow χ^2^ = 4.66, *P* = 0.19). The predictive effects of the validation cohort were comparable to those observed in the derivation cohort (Table 6).

## Discussion

The widespread use of broad-spectrum antibiotics has led to an increase in ESBL infections associated with a negative impact on patient outcomes.^1–10^ Early identification of patients at increased risk of ESBL is an important measure to ensure both time to optimal antibiotic therapy and consideration for isolation in hospitalized patients. Our results demonstrate that a simple clinical prediction tool based on institution-specific risk factors can be created to identify admitted patients most at risk of harboring ESBL BSI.

Our study showed an overall higher proportion of ESBL-producing isolates within our institution compared to previously reported data.^6–13^ Similar to other studies, we found that the predominant source of ESBL BSI was urinary followed by intra-abdominal.^6,7,9,11^ Specific antimicrobial classes have been independently associated with ESBL-producing organisms including fluoroquinolone and β-lactam agents, specifically oxyimino- β-lactams and third/fourth-generation cephalosporins.^6,12,13,28^ Our study found an association between ESBL production and use of third-generation cephalosporins, fluoroquinolones, β-lactam β-lactamase inhibitors, carbapenems, and uniquely trimethoprim-sulfamethoxazole. However, multivariate analysis did not find any single antimicrobial class to be an individual independent risk factor for ESBL production but rather found a general 30-day history of antimicrobial utilization as a risk factor.

Many studies have shown that patients with a history of urinary catheterization increased the risk of ESBL infection.^8,13,28^ Although ESBL patients in our cohort were more likely to have a history of urinary catheter insertion or invasive surgery, these were not identified as independent risk factors. However, a 30-day history of any invasive procedure was found to be a risk factor in multivariate analysis. Additionally, a review by Trecarichi *et al.* found that admission from long-term care facilities has been found to be strongly associated with non-nosocomial ESBL BSI.^8^ This similarity was demonstrated in our study as most of the infections were community-onset, and nearly one-third of patients with ESBL BSI were admitted from a skilled nursing facility.

Unique to our study was a history of diabetes being identified as an independent risk factor for ESBL BSI. In a study conducted by Rodriguez-Baño *et al.* describing clinical features of infections caused by ESBL in nonhospitalized patients, researchers found five independent risk factors associated with ESBL, among them diabetes mellitus.^29^ To our knowledge this is the only known study demonstrating diabetes mellitus as an independent risk factor. However, in contrast to our study, Rodriguez-Baño *et al.* found the emergent cause of ESBL to be urinary tract infection whereas only 6 patients were bacteremic.^29^ Our study suggests that diabetes mellitus may be considered a risk factor for community-onset ESBL BSI.

Demonstrated by our results and review of available literature, risk factors for ESBL seem to be institution-specific, which is valuable in identifying targets to influence clinical decision-making. As discussed by Trecarichi *et al.*, there is a large heterogeneity among studies markedly regarding species of Enterobacterales studied, acquisition of BSI, and study design which may explain the differences seen in epidemiology.^8^ Given that there is no standard recommendation for identification of ESBL, risk factors are likely influenced by institution-specific factors and increased intestinal colonization with multidrug-resistant organisms following recent use of antibiotic therapy.^20^ Additional considerations for patients presenting from the community with limited healthcare-associated contact may be necessary.

Given recent concerns about emerging carbapenem-resistant isolates, there is a pressing need for an accessible risk stratification tool that can be used at admission.^2^ Our study was conducted as a carbapenem-sparing antibiotic stewardship initiative for infections warranting empiric broad-spectrum therapy. Our scoring tool is unique to our inpatient population, which consists of primarily elderly patients residing at a skilled nursing facility and was constructed from variables that are readily available from the medical record at the time of admission, enhancing its practicality. Our study provided a good discrimination of risks in both independent derivation and validation cohorts with comparable ROC AUCs. These results are comparable to those reported by both Tumbarello *et al.* and Jones *et al.*, which conclude that the high specificity of a risk assessment tool could improve targeting patients at high risk for ESBL BSI.^20,21^ In another study conducted by Lee *et al.*, a 4-measure scoring algorithm was proposed to identify community-onset ESBL among patients in the emergency department.^19^ This algorithm included recent antimicrobial use or invasive procedure, nursing home resident, and frequent emergency department visits where designated scores of ≥2 retained a high sensitivity, high specificity, and good discrimination to identify patients at risk for ESBL.^19^ A threshold of ≥3 for result interpretation was chosen for our scoring tool to allow for other risk factors in the predication model to be considered aside from a history of ESBL. While implementation of newer technology decreases time to organism identification and resistance marker detection, the focus of our study remains for institutions with limited rapid diagnostics. The results of our study could aid in initiation of rational, empiric antimicrobial therapy by frontline providers while susceptibility results are pending, even in the absence of history of ESBL.

Our study has several limitations. First, the data set was a retrospective review which included a relatively low sample size and narrow time range. This may have underestimated the role of certain risk factors and limited the amount of independent risk factors analyzed in multivariate analysis. Additionally, applicability of these results to the pediatric population is limited. Since our data were exclusively analyzed from one tertiary hospital within a large integrated system, the generalizability of these results may be limited since risk factors are specific to the region or institution. Our study only interpreted ESBL production based off phenotypic testing without a genotypic confirmation, which could skew the results of confirmed ESBL isolates. Finally, interpretation of results in patients with a score ≤2 is limited. While sensitivity and NPV remain high for both cohorts, utilizing a score ≤2 may overcall ESBL risk and lead to overutilization of broad-spectrum antimicrobial therapy active against ESBL. Therefore, a more appropriate strategy for patients who score ≤2 using this assessment tool is to consider agents likely active against ESBL for patients at high risk for mortality, including those that are critically ill, with a plan to de-escalate once supporting microbiological data becomes available.

In conclusion, we have identified and analyzed independent risk factors associated with ESBL BSI. Using these data, a novel 7-point clinical predication tool was developed based on four easy-to-define variables readily available upon chart review. This prediction model may be used to reliably identify hospitalized patients that likely harbor ESBL upon organism identification. Proper use of this tool can minimize the time required to identify patients at risk for these organisms, allowing for decreased time to optimal therapy, and rapid infection prevention measures. Future efforts should focus on analyzing applicable ESBL BSI risk factors at other institutions given variability across regions and patient populations to devise a generalizable risk assessment tool that can be modified to fit institution-specific factors. This tool should be utilized as a carbapenem-sparing initiative, emphasizing the clinical application of such tools for empirical treatment decision-making as the utility in this setting requires further validation.
